# Site specific rates of mitochondrial genomes and the phylogeny of eutheria

**DOI:** 10.1186/1471-2148-7-8

**Published:** 2007-01-25

**Authors:** Karl M Kjer, Rodney L Honeycutt

**Affiliations:** 1 Rutgers University, Department of Ecology, Evolution, and Natural Resources, Blake Hall, 93 Lipman Drive, New Brunswick, New Jersey 08901-8524, USA; 2 Pepperdine University, Natural Science Division, 24255 Pacific Coast Hwy, Malibu, California 90263-4321, USA

## Abstract

**Background:**

Traditionally, most studies employing data from whole mitochondrial genomes to diagnose relationships among the major lineages of mammals have attempted to exclude regions that potentially complicate phylogenetic analysis. Components generally excluded are 3^rd ^codon positions of protein-encoding genes, the control region, rRNAs, tRNAs, and the ND6 gene (encoded on the opposite strand). We present an approach that includes all the data, with the exception of the control region. This approach is based on a site-specific rate model that accommodates excessive homoplasy and that utilizes secondary structure as a reference for proper alignment of rRNAs and tRNAs.

**Results:**

Mitochondrial genomic data for 78 eutherian mammals, 8 metatherians, and 3 monotremes were analyzed with a Bayesian analysis and our site specific rate model. The resultant phylogeny revealed strong support for most nodes and was highly congruent with more recent phylogenies based on nuclear DNA sequences. In addition, many of the conflicting relationships observed by earlier mitochondrial-based analyses were resolved without need for the exclusion of large subsets of the data.

**Conclusion:**

Rather than exclusion of data to minimize presumed noise associated with non-protein encoding genes in the mitochondrial genome, our results indicate that selection of an appropriate model that accommodates rate heterogeneity across data partitions and proper treatment of RNA genes can result in a mitochondrial genome-based phylogeny of eutherian mammals that is reasonably congruent with recent phylogenies derived from nuclear genes.

## Background

The class Mammalia provides a classic example of an adaptive radiation, characterized by a proliferation of lineages displaying a diverse array of ecomorphological specializations for feeding and locomotion [1]. Many additional biological attributes (e.g., behavior, physiology), coupled with this diversity in form and function, have allowed mammals to exploit a broad range of habitats worldwide. There are approximately 135 families of living mammals apportioned into 26 orders and two major subclasses, Prototheria and Theria, with the former subclass containing the order Monotremata (duck-billed platypus and spiny-anteaters) and the latter containing the infraclasses Metatheria (marsupials) and Eutheria (placentals), which are subdivided into 7 and 18 orders, respectively [2,3]. Lineage-specific rate heterogeneity in terms of morphological diversification [4] and molecular divergence [5-7] is a trademark of the various orders and families of mammals, especially within the Eutheria, and this has complicated efforts to resolve phylogenetic relationships among the higher categories of mammals.

Until relatively recently, most contributions to the "mammal tree of life," as it relates to phylogeny and classification, were made by functional morphologists and paleontologists [2,8-10]. More recent molecular efforts have resulted in confirmation of some previous hypotheses, the refutation of others, and the proposal of novel arrangements [10-13].

The most severe disagreements between morphology and molecules originated from studies based on mitochondrial genome sequences. For example, monophyly of Rodentia (the most speciose order of mammals) is based on a combination of dentition, skull morphology, soft anatomy, the postcranial skeleton, and the jaw mechanism [14], and early classifications never questioned the naturalness of this clade. Nevertheless, several early studies of nuclear genes [15-17] and mitochondrial genomes [18-20] argued that guinea pigs and presumably their relatives (hystricognath rodents from South America and Africa) were "not rodents," but represented a separate and more basal eutherian lineage, apart from muroid rodents (rats and mice). These same data challenged the monophyly of Glires, a group recognized on the basis of morphology [10,21] and containing the orders Lagomorpha (rabbits) and Rodentia, by suggesting a sister-group relationship between lagomorphs and primates [22]. The morphological placement of the order Xenarthra (armadillos, sloths, and anteaters) at the base of the eutherian radiation was also challenged, with mitochondrial data suggesting either the Erinaecidae [hedgehogs; [23]] or rodents at the base. In contrast to the morphology, xenarthrans were considered sister to a clade containing the orders Carnivora, Perrisodactyla (horses, rhinos, and elephants), Artiodactyla (pigs, antelope, deer, camels, etc.), and Cetacea (whales and dolphins) [24]. Two of the more startling results from the analysis of mitochondrial genomes included: 1) the placement the order Monotremata as sister to Metatheria, thus making the subclass Theria paraphyletic [25], and 2) a sister-group relationship between the anthropoid primates and Dermoptera (flying lemurs), thus rendering the order Primates paraphyletic [26]. Neither of these hypotheses is supported from either other molecular data or morphology [9,10,27-29].

More extensive studies employing greater taxon sampling as well as larger amounts of nucleotide sequence data from mitochondrial RNA (primarily rRNA) and/or nuclear genes [30-38] have resulted in higher levels of congruence with earlier morphological studies, including increased support for a more basal position of Xenarthra, the monophyly of Rodentia, Glires, and Primates, a monophyletic Theria, the Paenungulata (containing elephants, hyraxes, and sirenians), Tetytheria (elephants and sirenians), and Euarchonta (Scandentia, Dermoptera, Primates).

In contrast to recent studies employing primarily nuclear DNA sequences, a more recent study of whole mitochondrial genomes [26] failed to retrieve many of the well-supported clades identified by nuclear gene studies. Springer et al.'s [36] comparison of mitochondrial and nuclear gene sequences implied that mitochondrial data are less effective at resolving relationships at deeper nodes of the mammalian tree, and in many cases mitochondrial sequences failed to recover "benchmark clades," that are well-supported by both morphology and nuclear genes. In this particular comparison, nuclear genes apparently outperformed mitochondrial genomes because they evolve at a rate appropriate for resolving more divergent relationships among major lineages of mammals.

Unless mitochondrial genomes are evolving at rates where saturation becomes a problem at deeper nodes, one would expect the inclusion of analytical procedures that accommodate asymmetries observed for mtDNA [29,39-42], coupled with appropriate placement of the root of the eutherian tree [30,40,43] and increased taxon sampling [44-47], to result in mitochondrial phylogenies that are more congruent with the consensus reached by nuclear genes. For the most part, a consideration of these factors has improved more recent results, primarily because model-based analyses of more mitochondrial genomes were employed [41]. Nevertheless, as with earlier studies employing whole mitochondrial genomes, Reyes et al. [41] excluded several regions of the genome prior to analysis with a model that accommodated multiple rates of substitution. For instance, 3^rd ^codon positions, first positions involving leucine, and the control region are generally excluded to reduce homoplasy resulting saturation effects. The ND6 gene, encoded on the L-strand, is omitted because of presumed differences in constraints (e.g., base composition) relative to genes encoded on the H-strand. Finally, ribosomal genes (rRNAs) and transfer RNAs (tRNAs) are frequently left out, presumably because they are difficult to align.

It is our contention that exclusion of data is unnecessary if appropriate model-based analyses are employed. If fast evolving sites like 3^rd ^codon positions can be appropriately modeled, then there is little reason for excluding them from a likelihood-based analysis. Similarly, if rRNAs and tRNAs can be reasonably well aligned with secondary structure, we see little justification for excluding these characters. In this paper we provide an analysis of whole mitochondrial genomes from 89 mammalian taxa and investigate relationships among major lineages of eutherians. Except for the control region, which is difficult to align across highly divergent taxa, all sequences were used in an analysis employing a pseudoreplicate-generated, site-specific rate model, first proposed by Kjer et al. [48]. Our major goal is to evaluate the effectiveness of this model to negate *a prior *exclusion of potentially useful data, and we base our conclusions on comparison of results to more extensive studies based on a large panel of nuclear gene sequences and extensive taxon sampling.

## Results

**Figure 1:**
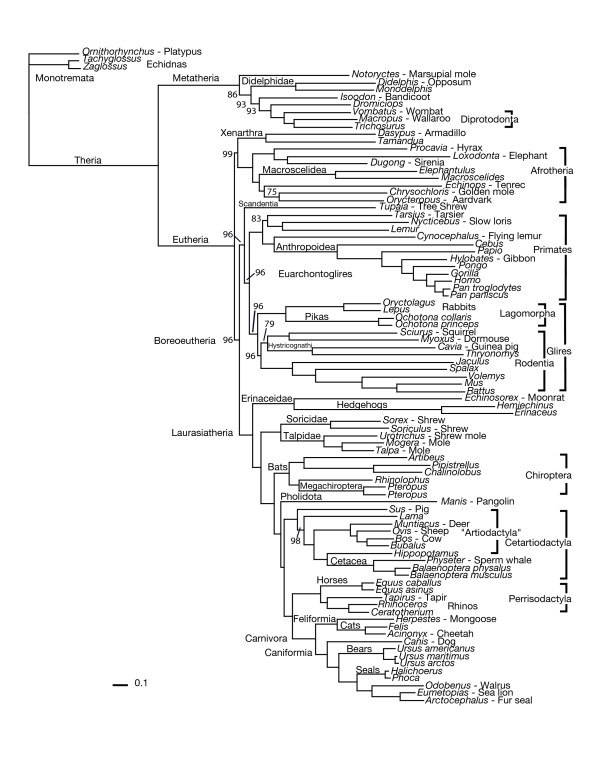
Most likely phylogram derived from the Bayesian Analysis (-ln 533753.675). Numerals indicate estimated posterior probability. These values are either placed on top of the node they represent (or with arrows pointing to the top of the internode) or directly to the left of the node. Nodes without numerals are supported at 100%. Higher taxa are indicated either on top of their representative internode, directly to the left of the node or to the right of the clade, and are delimited with brackets.

The annotated Nexus file consists of 14,740 nucleotides, includes 3,783 amino acid characters as well as additional taxa (not used in this analysis), and is available on Kjer's website [49]. The Nexus file on the website includes character set definitions ("charsets") that allow the user to identify and analyze single gene partitions, codon positions, and rate classes separately, and taxon set definitions ("taxsets") that allow the user to evaluate relationships among specific taxa. The most likely tree from the Bayesian analysis is shown in Fig. 1. This phylogeny reveals strong support for several major groups of eutherians including: 1) a monophyletic Afrotheria, a basal clade containing Proboscidea (elephants), Sirenia (manatees and dugongs), Hyracoidea (hyraxes), Macroscelidea (elephant shrews), Tubulidentata (aardvarks), Afrosoricidea (insectivore families Chrysochloridae or golden moles and Tenrecidae or tenrecs); 2) a monophyletic Xenarthra sister to Afrotheria; 3) Euarchontoglires represented by two major clades, one containing the Primates (including Anthropoidea, Tarsiformes, and Lemuriformes), with Dermoptera (flying lemurs) nested inside, and the other containing a monophyletic Glires (rabbits and rodents); 4) euarchontan order Scandentia (tree shrews) sister to the two major groups of Euarchontoglires; 5) Laurasiatheria containing a paraphyletic Eulipotyphyla (representing the insectivore families Erinaceidae and Soricidae, and Talpidae), Chiroptera (bats), Pholidota (pangolins), Cetartiodactyla (Artiodactyla and Cetacea or whales and dolphins), Perrisodactyla (horses, rhinos, tapirs), and Carnivora; 6) a sister-group relationship between Euarchontoglires and Laurasiatheria. In addition to these major clades, monophyly of Paenungulata (containing the orders Proboscidea, Sirenia, and Hyracoidea), Tethytheria (Sirenia and Proboscidea), and Cetartiodactyla (Artiodactyla and Cetacea) with cetaceans sister to hippo is strongly supported.

**Figure 2:**
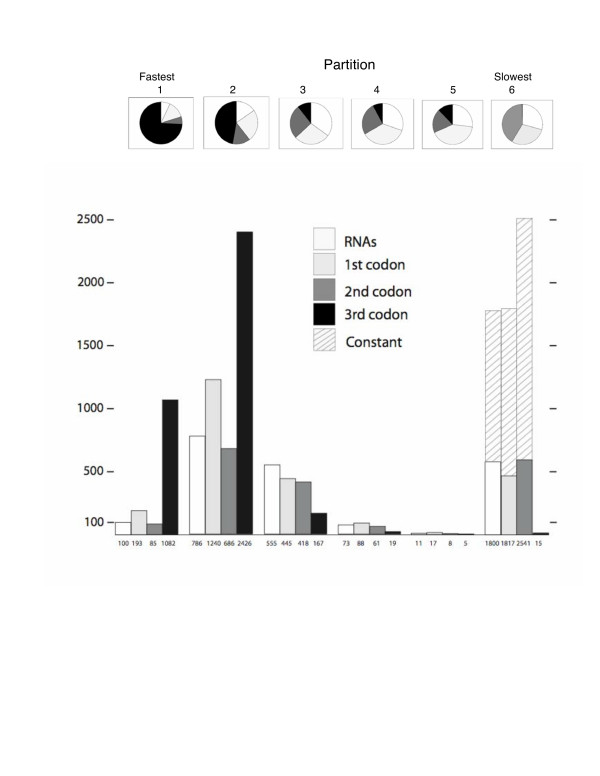
Rate Classes and Partition of Variable Sites – **Top**: A visualization with pie graphs of the proportion of sites in each rate-class partition that are RNAs (white), first codon positions (light grey), second codon positions (dark grey), and third codon positions (black). Rate classes are listed across the top, from fastest (class 1) to slowest (class 6). **Bottom**: A bar-graph visualization of the numbers of each of these classes among partitions, using the same color coding, as indicated in the key. Constant sites, found only in rate class six, are indicated with hatched bars. Raw numbers of each of the values in the bar graph are given below the bars. Fifteen sites from the origin belong in rate class 6, one in rate class 4, and two in rate class 3 (not shown).

Table 1 shows the number of characters in each class, the rescaled consistency indices (RC), the mean model parameters and rate classes associated with the six partitions. The RC values show that the rate classes are very different in terms of how well the data map onto the tree. The fastest rate class is C-T rich (80%), just as C-T transitions are the fastest substitution class while slower rate classes are much less biased in terms of nucleotide composition (Table 1). Among site rate variation is most pronounced at the slowest and the fastest rate classes. Figure 2 shows a characterization of the partitions in terms of codon position and RNAs. RNA sequences tended to be conservative, and in terms of rates were similar to 2^nd ^codon positions of protein-encoding genes. As expected, 3^rd ^codon positions were associated with the faster rate classes, although a portion of 3^rd ^positions evolved slowly (approximately 200 in rate classes 3–6). There were more parsimony-informative RNA characters (786), as well as first and second codon position characters (1928) in the "fast" rate class 2, than in rate class 6 (the slowest; 197 parsimony informative rRNA sites, and 258 parsimony informative 1st and 2nd codon sites). There were about the same number of variable RNA characters in rate class 6 (532) as there were second codon sites (543). We note that many 1^st ^and 2^nd ^codon sites are fast-evolving (2,206 in the fastest two rate classes), and 186 parsimony-informative (of 1800) RNA characters that have been discarded from other analyses are members of the slowest rate class, which is comparable to 131 (of 2541) parsimony-informative second codon positions in rate class 6.

**Table 1:** Mean model parameters and six character partitions and rate classes

Partitions	1	2	3	4	5	6
Character	1460	5138	1585	241	41	6275
Const.	0	0	0	0	0	4719
Inform	1460	5138	1585	241	41	459
RC	0.02	0.048	0.172	0.332	0.448	0.818
r(A<->C)	1E-05 ± 4E-05	0.26 ± 0.001	0.131 ± 0.005	0.113 ± 0.011	0.060 ± 0.026	0.124 ± 0.008
r(A<->G)	0.833 ± 0.107	0.444 ± 0.006	0.307 ± 0.010	0.235 ± 0.107	0.200 ± 0.066	0.299 ± 0.012
r(A<->T)	0.008 ± 0.007	0.042 ± 0.001	0.092 ± 0.004	0.129 ± 0.011	0.066 ± 0.029	0.110 ± 0.006
r(C<->G)	5E-05 ± 8E-05	0.021 ± 0.001	0.063 ± 0.005	0.118 ± 0.015	0.224 ± 0.070	0.128 ± 0.009
r(C<->T)	0.137 ± 0.101	0.280 ± 0.006	0.341 ± 0.010	0.284 ± 0.101	0.230 ± 0.064	0.274 ± 0.011
r(G<->T)	0.022 ± 0.003	0.186 ± 0.003	0.066 ± 0.004	0.121 ± 0.013	0.221 ± 0.071	0.065 ± 0.005
pi(A)	0.18 ± 0.04	0.44 ± 0.00	0.31 ± 0.01	0.32 ± 0.02	0.47 ± 0.09	0.25 ± 0.00
pi(C)	0.44 ± 0.02	0.29 ± 0.00	0.21 ± 0.01	0.21 ± 0.01	0.24 ± 0.05	0.23 ± 0.00
pi(G)	0.03 ± 0.01	0.06 ± 0.00	0.18 ± 0.01	0.19 ± 0.01	0.09 ± 0.03	0.21 ± 0.00
pi(T)	0.36 ± 0.02	0.21 ± 0.00	0.30 ± 0.01	0.28 ± 0.02	0.21 ± 0.04	0.32 ± 0.01
alpha	0.623 ± 0.170	0.932 ± 0.012	3.361 ± 0.260	42.83 ± 5.770	27.39 ± 615.515	0.879 ± 0.100
m	5.76 ± 0.41	1.17 ± 0.11	0.15 ± 0.01	0.11 ± 0.01	0.31 ± 0.40	0.01 ± 0.00

"Character" refers to the number of characters in a partition. "Const." is the number of constant (invariant) sites, and "Inform" is the number of parsimony informative sites. "RC" is the rescaled consistency index. The next six lines are the values from the rmatrix, followed by the percentages of each of the nucleotides. "Alpha" is the shape parameter from the gamma distribution, and "m" refers to the relative rates among partitions. Rates increase from classes 1 to 6.

## Discussion

This analysis shows that third codon positions, redundant first codon (leucine) positions, the ND6, and the RNA genes can be included in a combined model-based analysis without drastically contradicting the general consensus from previous molecular studies. In fact, all benchmark clades for eutherian mammals that could be compared to the list provided by Springer et al. [36] were retrieved in our analysis and received high support. These benchmark clades include (all posterior probabilities 100): 1) Carnivora (Feliformia + Caniformia); 2) Cetacea (toothed whales and dolphins + baleen whales); 3) Cetartiodactyla (Artiodactyla + Cetacea); 4) Chiroptera (bats); 5) Diprotodontia (wombats, wallaroos, and brush-tailed possums); 6) Paenungulata (hyrax + elephants and Sirenia); 7) Perrisodactyla (rhino and tapir + horses); 8) Rumantia (bovines, sheep, deer); and 9) Xenarthra (armadillo + tamandua). The mitochondrial genome-based phylogeny shown in Fig. 1 is congruent with previous nuclear gene studies [32-34,50] in several respects. Although placement of the root varies among studies [51], the nuclear gene studies and our study place the groups Afrotheria and Xenarthra at the base of the eutherian phylogeny followed by a sister-group relationship between the monophyletic groups Euarchontoglires and Laurasiatheria (collectively called the Boreoeutheria). Several other monophyletic groups appear to be well-supported and congruent between our mtDNA and previous nuclear DNA studies including Paenungulata (Hyracoidea, Sirenia, and Proboscidae), Cetartiodactyla (Artiodactyla and Cetacea), Chiroptera, and Glires (Lagomorpha and Rodentia).

Although several groups are identified by both our whole mitochondrial genome analysis and nuclear genes, not all of these molecularly-defined groups are necessarily congruent with morphological data. For instance, some morphological studies support a monophyletic Archonta containing the euarchontans as well as Chiroptera [9,10], and although a relationship between the orders Artiodactyla and Cetacea has support from morphology, a sister-group relationship between Cetacea and the family Hippopotamidae (hippos) denoted by both nuclear genes and mitochondrial genomes [52] is supported by some [53] but not all morphological analyses [54,55]. Some earlier morphological comparisons [9], but none of the molecular data, support Volitantia, a group containing Chiroptera and Dermoptera. More recent molecular studies, including the one presented here, have indicated paraphyly for the chiropteran suborder Microchiroptera with the family Rhinolophidae grouping closer to the Megachiroptera, a clade containing non-echolocating taxa [56-58], and this is not corroborated by morphological data.

Our phylogenetic results are similar to those presented by Reyes et al. [41], which was based on a GTR+I+G Bayesian analysis that excluded RNAs, ND6, and redundant codon positions. Gibson et al. [39] also showed that there were lineage and gene specific biases of C and T compositions, and performed an analysis with a model that reduced the character complexity of these nucleotides to Y, creating a three-state model. While Gibson et al. [39] included RNAs, they also excluded third codon positions and the ND6, resulting in a dataset of 7,402 sites. While we agree with the corrections proposed by both Gibson et al. [39] and Reyes et al. [41] in reducing the influence of homoplastic and biased characters, our approach differed in including a site specific rate model that rendered noisy sites less influential at deeper nodes, while retaining them as characters toward the tips of the tree. Our matrix is nearly twice the size of the largest previous analyses. In performing the pseudoreplicate reweighting, the noisiest sites are presumably identified and accommodated in a model. Many different partitions, including those that were excluded by others, can be explored by downloading the Nexus file and including specific "charsets" such as the ND6. For example, a parsimony analysis of the ND6 gene results in the recovery of therians, metatherians, eutherians, anthropoid primates (in the same order as the combined analysis), whales, and carnivores, among other groups (not shown). Clearly, the ND6 contains some non-random signal, including 26% of its 535 nucleotides in rate class 6 (the slowest).

The trees in our analysis of the combined data differ from others in the placement of Xenarthra; ours with Afrotheria (Fig. 1), supporting a northern-southern hemisphere split, and Gibson et al. [39] and Reyes et al. [41] with Euarchontoglires. Note, both this analysis and the analysis of Gibson et al. [39] compensate for the large number of homoplastic C-T transitions but in different ways. Kriegs et al. [59], using retrotransposed elements (which they suppose to be "homoplasy free"), supported Xenarthra as the sister taxon of the rest of Eutheria. While we agree that the two retrotransposed elements supporting this relationship are exceedingly strong characters, we prefer to consider the independent loss of these in the sloth and the armadillo as "possible but unlikely." The rest of Kriegs et al.'s [59] conclusions are supported by our analysis. The placement of *Manis *(pangolin) also differs between this hypothesis and Gibson et al. [39] and Reyes et al [41]. Although we show 100% posterior probability for our hypothesis, we also note the exceedingly short branch length of the internode placing *Manis *as the sister taxon to (Cetartiodactyla(Perissodactyla(Carnivora))). Lewis et al. [60] describe conditions under which Bayesian posterior probabilities may be inflated, and we have not corrected for potentially inflated support for our placement of both *Manis *and Xenartha. The placement of Xenarthra with Afrotheria and the position of *Manis *in our phylogenetic hypothesis are congruent with Hudelot et al. [31], who used a 7-state doublet model to accommodate paired RNA sites. Similarities between this study and Hudelot et al [31] could be attributed to the inclusion of RNAs in both studies, while differences are more likely due to differences between models.

Finally, the mitochondrial genome data, even after inclusion of all sequences and a model that incorporates multiple rate classes, reveal several anomalies that are not congruent with recent nuclear gene phylogenies. Some particular anomalies appear to be inherent to all mitogenomic analyses [26,28,39,41], regardless of either taxon sampling or the phylogenetic methods employed. Rather than a monophyletic Primates, as revealed by nuclear genes, our analyses as well as previous mitochondrial phylogenies indicate a paraphyletic Primates with the order Dermoptera (flying lemurs) sister to anthropoid primates (monkeys, lesser and great apes) to the exclusion of the other primate lineages such as tarsiers and prosimians (lemurs). Monophyly of the insectivore group Eulipotyphla, containing the families Erinaceidae, Soricidae, and Talpidae, is supported by nuclear gene phylogenies [32-34,61] but not by mitochondrial data, which in our case indicates eulipotyphlan diphyly with the Erinaceidae (hedgehogs) at the base of the Laurasiatheria clade. The order Scandentia (tree shrews) is generally considered sister to either Dermoptera or Primates based on recent molecular and morphological data [10,33,34,50], whereas mitogenomic analyses place scandentians at the base of Euarchontoglires. Additionally, mitochondrial data support a monophyletic Tethytheria (elephants and manatees), whereas the more recent nuclear studies [34] do not, and although recent molecular data [62] place marsupial moles (*Notoryctes*) as part of a monophyletic group (Australidelphia) confined to Australia, our analysis places them basal to other lineages of Metatheria.

Persistent incongruence between mitochondrial and nuclear gene phylogenies relative to the placement of some mammalian lineages may have more than one explanation. Long-branch attraction is often used as an explanation for misplacement of taxa [63,64], and many of the ambiguous placements involve lineages with longer branches (Fig. 1). As indicated by Bergsten [63], outgroups can often influence placement of ingroup taxa, which may be the case for the position of the marsupial mole. Increased taxon sampling and the incorporation of maximum likelihood models for mitogenomic analyses [63] did remove the Erinaceidae from a basal position in the placental phylogeny to one associated with the Laurasiatheria. Nevertheless, these modifications do not result in a monophyletic Eulipotyphla, as suggested by nuclear genes. In the case of the placement of Dermoptera, there is no apparent reason to consider this as the result of either long branches or branch support from character partitions in the higher rate classes. Schmitz et al. [28] suggested an association between demopteran and anthropoid primate mitochondrial sequences being the result of similarities in nucleotide and amino acid composition. However, Hudelot et al. [31] recovered a monophyletic primates with their doublet model, with the flying lemur as its sister taxon, despite similarities in nucleotide composition at third positions between the flying lemur and Anthropoidea. Finally, if these areas of incongruence are the result of similarities in base composition, covariotide/covarion effects, or some other source of heterogeneity [64], it may very well be that no existing model adequately corrects for all anomalies observed for the mammalian mitochondrial genome.

## Conclusion

Although some incongruence still remains between phylogenies derived from mitochondrial and nuclear sequences, our results indicate that the exclusion of data is not necessary for an effective reconstruction of eutherian relationships (although we still excluded the control region and unalignable RNA sites). Rather, selection of an appropriate model that accommodates rate heterogeneity across data partitions and proper treatment of RNA genes can yield information highly congruent with more extensive nuclear sequences, even when addressing the deepest nodes of the eutherian phylogeny. And while we are using "expected" clades to support our conclusions, we note that we are not using phylogenetic expectations as a rationale to *exclude *data, as is often the case, but rather to retain data. Arguments to retain data should be met with a lower burden of proof than arguments to exclude data.

## Methods

Mitochondrial genomes were downloaded from GenBank. A Nexus file was constructed, with each block in the file corresponding to either one gene or a block of data between 100–150 nucleotides for manually aligned rRNAs (the number of nucleotides that are visible on one computer screen without scrolling). Nucleotides between genes were manually aligned, and unaligned regions were placed between brackets (which eliminates them from the dataset, while retaining them for visual inspection). Ribosomal RNAs and tRNAs were aligned manually with reference to secondary structure, according to recommendations of Kjer [65] and Gutell et al. [66].  Models for rRNA secondary structure came from the Comparative RNA Web (CRW) Site [67]. The control region was eliminated. All other genes and codon positions were included. Genes coded in the reverse strand were reversed and complemented.

A site specific rate model was constructed according to Kjer et al. [48]. Briefly, a fast heuristic bootstrap analysis, with 1000 replicates, was completed in PAUP, having saved one tree per replicate. The characters were then separated into 6 discrete rate classes by first selecting the "reweight characters" option in PAUP, according to the "best" CI from among the 1000 bootstrap-generated trees. By selecting "view character weights," and editing the resultant output, we constructed a file in Microsoft Excel that was sorted according to the weights, and then re-imported into the Nexus file to construct 6 partitions or "charsets" from fastest to slowest. These charsets were then used in a partitioned Bayesian analysis, with each partition free to vary according to its own GTR + gamma model.

Each Bayesian analysis was performed with 3 hot and one cold chain. Burnin periods were graphically visualized from the .p files from MrBayes and viewed in Excel. The first set of two independent Bayesian analyses was run for 7.5 million generations in MrBayes 3.0 [68]. Since the likelihood scores from these two chains were not the same, another pair of analyses was conducted in MrBayes 3.1 [68]. This analysis was terminated with a power-failure after 5 million replicates. However, these runs had stabilized on the same likelihood plateau, which was the same as the better of two earlier runs of 7.5 million. Therefore, after discarding the burnin, trees from all three optimal analyses were pooled into a single tree file, from which a majority rule consensus was used to visualize posterior probability values. The best tree was visualized with Treeview [69], and the likelihood phylogram was exported as a pict file for modification.

## Authors' contributions

KMK collected genome sequences from GenBank, aligned sequences, and performed initial analyses. RLH provided a detailed comparison of the new phylogeny to previous phylogenetic hypotheses for mammalian relationships and interpreted results relative to ideas concerning the evolution of mammals.
